# Nomogram to Predict Internal Mammary Lymph Nodes Metastasis in Patients With Breast Cancer

**DOI:** 10.3389/fonc.2019.01193

**Published:** 2019-11-08

**Authors:** Xinhua Xie, Zhenchong Xiong, Xing Li, Xiaojia Huang, Feng Ye, Hailin Tang, Xiaoming Xie

**Affiliations:** Laboratory of Oncology in South China, Department of Breast Oncology, State Key Collaborative Innovation Center for Cancer Medicine, Sun Yat-sen University Cancer Center, Guangzhou, China

**Keywords:** breast cancer, IMLN metastasis, nomogram, pALN stage, recurrence-free survival

## Abstract

**Background:** Numerous studies have showed that internal mammary lymph node (IMLN) metastasis is an important adverse prognostic factor in patients with breast cancer (BC), however, there are no available prediction model for the preoperative diagnosis of IMLN metastasis.

**Methods:** Data from 102 breast cancer patients treated with IMLN operation were used to establish and calibrate a nomogram for IMLN status based on multivariate logistic regression. Prediction performance of this model was further validated with a second set of 50 patients with BC. Discrimination of the predict model was assessed by the C-index, and calibration assessed by calibration plots. Moreover, we conducted the decision curve analysis (DCA) to evaluate the clinical value of the nomogram. Finally, the survival status of patients in different risk groups based on nomogram were also compared.

**Results:** The final multivariate regression model included tumor location, lymph vascular invasion (LVI), and pathological axillary lymph node stage (pALN stage). A nomogram was developed as a graphical representation of the model and had good calibration and discrimination in both sets (with C-index of 0.86 and 0.83 for the training and validation set, respectively). Moreover, the DCA showed the clinical usefulness of our constructed nomogram. False negative (FN) in low risk group classified by nomogram (FN-LR-nomogram) did not significantly impact adjuvant treatment decision making, and more importantly, patients with FN-LR-nomogram had recurrence-free survival equivalent to patients with pathologically ture negative in low risk group classified by nomogram (TN-LR-nomogram).

**Conclusions:** As a non-invasive prediction tool, our nomogram shows favorable predictive accuracy for IMLN metastasis in patients with BC and can serve as a basis to integrate future molecular markers for its clinical application.

## Introduction

Numerous studies have showed that internal mammary lymph node (IMLN) metastasis has similar prognostic value as axillary lymph nodal involvement, and IMLN status is one of the most important prognostic factors in patients with breast cancer (1, 2). The earlier studies showed that one third of breast cancer patients had IMN involvement, with a higher risk in patients with medially placed tumors and/or positive axillary lymph nodes (2, 3). Due to the drainage areas of the IMLNs and the axillary lymph nodes are regarded as the first nodal stations for lymphatic node drainage, many studies have been conducted based on lymphoscintigraphy and approximately one fifth of internal mammary sentinel nodes are found to be pathologic (4). In addition, results from randomized trials on post-mastectomy irradiation have provided high levels of evidence that local-regional tumor control might improve patients' long-term survival, however, the survival benefit might be offset by radiotherapy-associated heart disease (1, 2). Although minimally invasive technology such as video-assisted thoracoscopic surgery (VATS) has been successfully performed for IMLN after breast surgery with less complication, there are still 60% of patients are reported to be IMLN non-metastasis and these patients can be deemed to be over-treatment (5).

Therefore, it is vital to seek an effective method to assess IMLN status accurately and then select high-risk candidate patients for VATS or radiotherapy, while low-risk patients should be avoided IMLN treatment. Then, the diagnosis and treatment of IMLN involvement has become an important topic in the management of breast cancer. Several nomograms have been developed in the field of lymph node metastasis prediction, including one for predicting the risk of positive lymph nodes before operation in colorectal cancer (6). However, there is no prediction model constructed for IMLN status in breast cancer. Then, in the current study, we tried to develop and validate a user-friendly nomogram based on our cancer center data to predict individual probability of positive IMLNs based on clinicopathological risk factors.

## Materials and Methods

### Patients

Study protocol was approved by the institutional review boards of Sun Yat-Sen University Cancer Center (SYSUCC). Consecutive patients histologically diagnosed as breast cancer between January 2000 and March 2017 in SYSUCC were retrospectively reviewed. Inclusion criteria were as follows: (1) received mastectomy and IMLN surgery; (2) female; (3) pathological diagnosed as invasive ductal carcinoma (IDC) or invasive lobular carcinoma (ILC). Patients without enough data could be extracted. Every enrolled patient was randomly allocated as “training” or “validation” at the ratio of 2:1 and 75% of participants were selected as the training cohort. The remaining patients were grouped as the validation cohort. All enrolled patients had mastectomy and we conducted the IMLN surgery according to patients' pre-operative examination IMLNs dissection were conducted by video-assisted thoracoscopic surgery (VATS) for most patients. Of course, primary tumors in the inner quadrant are more likely to undergo IMLNs dissection, however, our case data were collected retrospectively and continuously.

Clinical characteristics collected for subsequent analysis included age, tumor location, pathological tumor stage, number of positive axillary lymph nodes, estrogen receptor (PR), progesterone receptor (PR), and human epidermal growth factor receptor-2 (HER2) status, Neoadjuvant chemotherapy (NAC) received or not, and imaging-reported IMLN status. The clinical stages were classified according to the AJCC TNM staging system (7th edition). ER and PR positivity were defined by the presence of more than 1% positive cells based on immunohistochemistry results, while HER2 positive was defined as “3+” in immunohistochemical test or “positive” in HER2 fluorescence *in situ* hybridization test. In our study, positive axillary lymph nodes (ALN) were defined after ALN dissection or sentinel node with complementary ALN dissection pathologically. Moreover, the detection methods for imaging-reported IMLN involvement status included computed tomography (CT) and/or Magnetic resonance examination (MR). Imaging scans were reviewed by two radiologists with >10 years of experience, who were blinded to clinical characteristics and post-operative pathological findings. Patients with IMLN of >1 cm and/or clusters of ≥3 lymph nodes were identified as clinically LN-positive, and patients without enlarged or clustered lymph nodes were regarded as clinically LN-negative. Any disagreement was resolved by consultation. Patients were censored from follow-up for survival at June 30, 2018, and from followup for relapse at the latest known recurrence-free date before or at June 30, 2018.

### Statistical Analysis

The primary outcome for this study was the likelihood of positive lymph nodes in internal mammary area following IMLN surgery. Multivariate logistic regression analysis was used to test the association between clinicopathological variables and the likelihood of IMLN metastasis. Coefficients for each variable and the constant in the equation were generated based on multivariate analysis. A nomogram was constructed to be a graphic representation of the prediction model with the R software.

Model performance was quantified in both the modeling group and the validation group with respect to discrimination and calibration. Discrimination was assessed by calculating the concordance index (c-index). Calibration was studied graphically after grouping patients into decile with respect to their predicted probabilities and plotting the mean predicted probabilities against the mean observed probabilities. Bootstrapping was applied to calculate 95% confidence intervals. Overall fit of the model was evaluated using the Hosmere-Lemeshow goodness of fit test for logistic regression. Reported *P*-values are two-sided with alpha 5%. Decision curve analysis for the nomogram was also evaluated. After obtaining the risk scores from the nomogram, we defined an optimal risk score cutoff value and patients were then classified into low- or high-risk groups accordingly. In low risk nomogram patients, the consistency between two breast oncology medical reviewers' recommendations and actual therapy were calculated in both the false negative (FN) and true negative (TN) groups separately, then we compared the clinical-pathological factors in the two groups.

Statistical analyses were performed using the statistical packages SPSS (SPSS for Windows, version 22.0, SPSS Inc., Chicago, IL) and R software (version 3.4.1; https://www.r-project.org/). The packages of R used in this study are as follows: “rms,” “Hmisc,” and “Dca.R.” The conventional 2-sided tests, and a significance level of 0.05 were used in all analyses. We compared the two groups using the χ^2^ test or Fisher exact test for categorical variables.

## Results

### Clinical Characteristics

**Figure 1:**
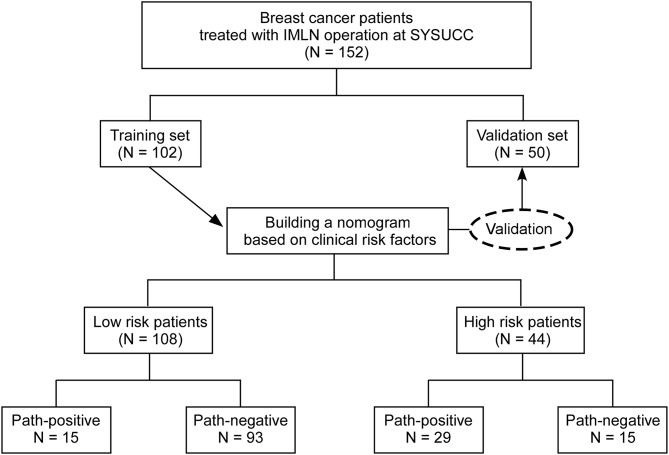
Schematic results of constructed nomogram and final IMLN pathology.

A total of 152 female patients with primary invasive breast cancer (102 in the training cohort and 50 in the validation cohort) fulfill the inclusion criteria and were enrolled to develop and validate our predictive nomogram model (Figure 1). Among the 152 patients, only 4 patients are diagnosed as ILC, while others are IDC. All the enrolled patients underwent IMLN operation. Patient and tumor characteristics are shown in Table 1. No significant difference was observed between the training cohort and validation cohort regarding the clinicopathological factors analyzed. There were nine patients received NAC. The candidate positive rate of imaging-reported IMLN was only 17.1% (26/152), while the actual positive detection rate for IMLN was 28.9% (44/152). Among the ALN negative patients, 9 patients were IMLN positive pathologically. Similarly, among the imaging-reported IMLN negative patients, 35 patients were IMLN positive pathologically. A total of 489 IMLNs were examined and the average number of removed IMLNs per patients were 3. The average examined IMLN did not differ in the two cohorts (Table 1).

**Table 1:** Clinicopathological characters in training and validation cohorts.

**Characteristics**	**Training cohort** **(*n* = 102)**	**Validation cohort** **(*n* = 50)**	***P***
Age			0.228
≤ 40	32 (31.4)	11 (22.0)	
>40	70 (68.6)	39 (78.0)	
Tumor size			0.778
T1	38 (37.3)	16 (32.0)	
T2	56 (54.9)	29 (58.0)	
T3	8 (7.8)	5 (10.0)	
Tumor location			0.796
UIQ	42 (41.2)	21 (42.0)	
LIQ	13 (12.7)	4 (8.0)	
Central	6 (5.9)	2 (4.0)	
UOQ	35 (34.3)	21 (42.0)	
LOQ	6 (5.9)	2 (4.0)	
ER			0.203
Negative	36 (35.3)	23 (46.0)	
Positive	66 (64.7)	27 (54.0)	
PR			0.101
Negative	33 (32.4)	23 (46.0)	
Positive	69 (67.6)	27 (54.0)	
Her2			0.282
Negative	75 (73.5)	32 (64.0)	
Positive	19 (18.6)	15 (30.0)	
Others	8 (7.8)	3 (6.0)	
LVI			0.824
Negative	80 (78.4)	40 (80.0)	
Positive	22 (21.6)	10 (20.0)	
pALN stage			0.963
N0	48 (47.1)	25 (50.0)	
N1	31 (30.4)	14 (28.0)	
N2	13 (12.7)	7 (14.0)	
N3	10 (9.8)	4 (8.0)	
NAC received			0.482
Yes	7 (6.9)	2 (4.0)	
No	95 (93.1)	48 (96.0)	
Imaging-reported IMLN status			0.103
Negative	81 (79.4)	45 (90.0)	
Positive	21 (20.6)	5 (10.0)	
Removed pIMLN	3.5 ± 2.0	3.2 ± 2.6	0.405
pIMLN status			0.857
Negative	72 (70.6)	36 (72.0)	
Positive	30 (29.4)	14 (28.0)	

*ER, estrogen receptor; PR, progesterone receptor; Her2, human epidermal growth factor receptor-2; UIQ, upper inner quadrant; LIQ, lower inner quadrant; UOQ, upper outer quadrant; LOQ, lower outer quadrant; LVI, lymphvascular invasion; pALN, pathological axillary lymph node; pIMLN, pathological internal mammary lymph node; NAC, Neoadjuvant chemotherapy*.

### Predictive Nomogram for the Probability of IMLN Metastases

**Figure 2:**
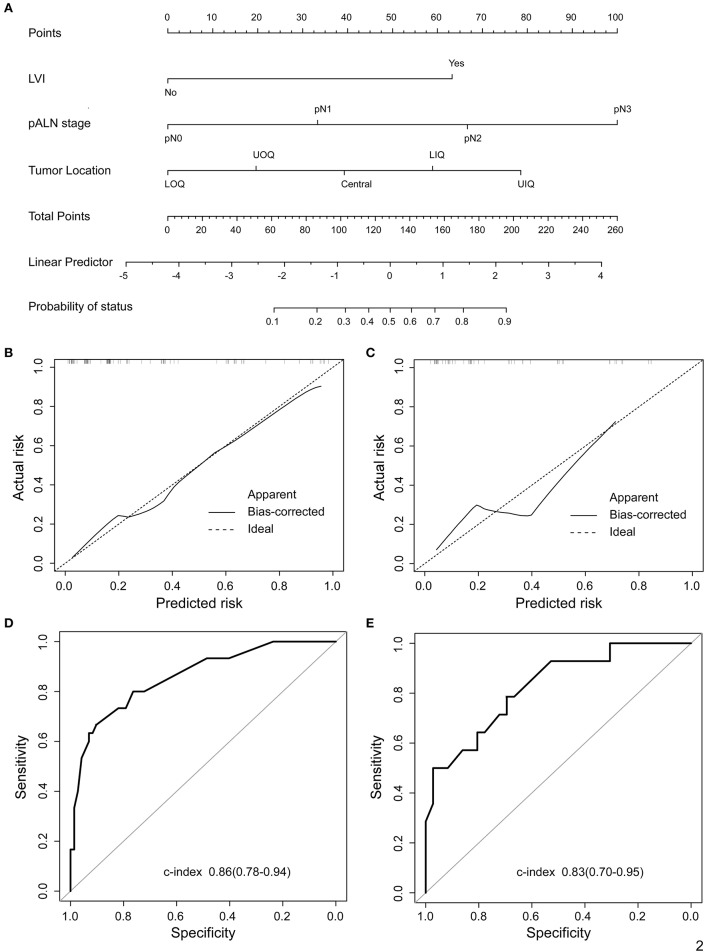
Proposed nomogram to predict the probability of IMLN metastasis after mastectomy in patients with breast cancer. **(A)** Nomogram was build to predict IMLN status for BC patients, with the tumor location, LVI and pALN stage incorporated. Calibration plots for nomogram model in **(B)** training cohort and **(C)** validation cohort. The dashed line (the 45-degree line) represents a perfect prediction nomogram, and the black solid line represents the observed nomogram, of which a closer fit to the dashed line means a better prediction model. Plots **(D)** and **(E)** show the ROC curves of the constructed nomogram in the training and validation cohorts, respectively. UIQ, upper inner quadrant; LIQ, lower inner quadrant; UOQ, upper outer quadrant; LOQ, lower outer quadrant.

In univariate analysis of the training cohort (Table 2), IMLN metastases were significantly correlated with tumor location, tumor stage, LVI, and pathological axillary lymph node stage (pALN stage). In multivariable analysis of the training cohort (Table 2), IMLN metastasis were significantly correlated with tumor location, LVI and pALN stage. On the basis of the multivariable logistic regression of the training cohort, a nomogram incorporating the significant risk factors was set up to predict the involvement probability of IMLN (Figure 2A). A total score was calculated using tumor location, LVI and pALN stage. Each value of these risk factors was allocated a score on the point scale axis. For example, tumor location at lower outer quadrant (LOQ) was 0 point and upper inner quadrant (UIQ) was 79 points. Interestingly, the allocated scores of pathological ALN stage showed that patients with pALN3 and pALN2 might be more likely to be IMLN involvement than patients with pALN0 and pALN1. A total score could be easily calculated by adding each single score and located this sum on the total point scale axis. Then draw a vertical line downwards from this point and identify the IMLN metastasis risk probability after mastectomy.

**Table 2:** Univariate and multivariate analysis for factors associated with internal mammary lymph node (IMLN) metastasis.

**Characteristics**	**Univariate analysis**		**Multivariate analysis**	
	**OR** **(95% CI)**	***P*-value**	**OR** **(95% CI)**	***P*-value**
Age				
≤ 40	1			
>40	1.207 (0.392–3.712)	0.743		
Tumor size				
T1	1		1	
T2	1.372 (0.516–3.651)	0.527	2.183 (0.553–8.628)	0.265
T3	26.250 (2.807–25.523)	0.004	12.114 (0.550–26.687)	0.114
Tumor location				
UIQ	1		1	
LIQ	1.543 (0.438–5.439)	0.5	0.864 (0.144–5.181)	0.873
Central	1.800 (0.322–10.055)	0.503	0.146 (0.008–2.778)	0.201
UOQ	0.300 (0.096–0.936)	0.038	0.098 (0.020–0.486)	0.004
LOQ	0.360 (0.038–3.374)	0.371	0.051 (0.001–3.823)	0.177
ER				
Negative	1			
Positive	0.919 (0.378–2.231)	0.851		
PR				
Negative	1			
Positive	0.939 (0.379–2.324)	0.891		
Her2				
Negative	1			
Positive	1.187 (0.399–3.533)	0.758		
Others	1.543 (0.338–7.037)	0.575		
LVI				
Negative	1		1	
Positive	12.571 (4.179–37.817)	0.000007	12.571 (4.179–37.817)	0.000007
pALN stage				
N0	1		1	
N1	2.864 (0.903–9.086)	0.074	5.399 (1.224–23.812)	0.026
N2	8.167 (2.042-12.654)	0.003	9.458 (1.711–22.293)	0.010
N3	28.000 (14.770–44.371)	0.000224	53.219 (12.772–68.245)	0.022
NAC received				
No	1			
Yes	0.957 (0.175–5.229)	0.960		
Imaging-reported IMLN status				
Negative	1			
Positive	1.261 (0.451–3.524)	0.658		

*ER, estrogen receptor; PR, progesterone receptor; Her2, human epidermal growth factor receptor-2; UIQ, upper inner quadrant; LIQ, lower inner quadrant; UOQ, upper outer quadrant; LOQ, lower outer quadrant; LVI, lymphvascular invasion; pALN, pathological axillary lymph node; pIMLN, pathological internal mammary lymph node; NAC, Neoadjuvant chemotherapy*.

### Validation for Predictive Accuracy of the Nomogram

**Figure 3:**
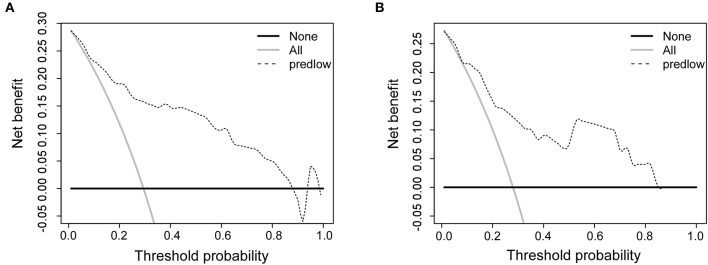
Decision curve analysis comparing the net-benefit of using the nomogram (black dashed line) depicted in **(A)** training cohort and **(B)** validation cohort. Black solid line: net benefit when all breast cancer patients are considered as not having the IMLN metastasis; gray solid line: net benefit when all breast cancer patients are considered as having the event. The ideal model is the model with the highest net benefit at any given threshold.

The calibration curves for IMLN metastasis showed good calibration (Figures 2B,C) and predicted well in both the training cohort and the validation cohort (c-index: 0.86 for training cohort and 0.83 for validation cohort; Figures 2D,E). In addition, we conducted the decision curve analysis for the constructed nomogram model (Figures 3A,B). The plot showed that, for predicted probability thresholds between 0 and 84%, model-based decision showed a more net benefit than either the treat-none-patients scheme or the treat-all scheme.

### Performance and Clinical Significance of the Novel Nomogram

After the enrolled patients were classified into low- or high- risk group by the nomogram model, we found 15 patients in low risk group are IMLN positive pathologically, while 15 patients in high risk group are identified to be IMLN negative (Figure 1). To further assess the potential adverse impact of false negative (FN) in low risk group classified by nomogram (FN-LR-nomogram) on adjuvant treatment strategy decision making, two breast oncologists performed a blinded review of clinical and pathological data (not include pIMLN status) from FN-LR-nomogram patients and matched ture negative (TN) patients in low risk group classified by nomogram (FN-LR-nomogram). The detailed data are shown in Supplementary Table 1. In the FN-LR-nomogram and TN-LR-nomogram groups, all risk factors were well-matched. Based on the data provided, the two reviewers made blinded adjuvant treatment recommendations for all LR-nomogram patients on the assumption that the patient's IMLN was negative pathologically. The treatments actually received were then compared with the recommendations of the two reviewers. There were no significant difference between the recommended treatment strategies and the treatment actually received in both the FN-LR-nomogram and TN-LR-nomogram groups (Table 3). Consistency between the reviewer #1 recommendation and the actual treatment strategy was 66.7% in the FN-LR-nomogram group and 80% in the TN-LR-nomogram group. Agreement between the reviewer #2 recommendation and the actual treatment strategy was 80% in the FN-LR-nomogram group and 77.3% in the TN-LR-nomogram group. Agreement between reviewer #1 and reviewer #2 was 80% in the FN-LR-nomogram group and 66.7% in the FN-LR-nomogram group. Details of the actual treatments received, and the treatments recommended in the blinded review are presented in Supplementary Figure 1.

**Table 3:** Concordance between actual treatment and blind review treatment recommendations, Group 1 (false negative nomogram) and Group 2 (true negative nomogram).

	**Group 1**	**Group 2**	***P***
Actual and #1	66.7% (10/15)	73.3% (11/15)	0.873
Actual and #2	80% (12/15)	80% (12/15)	0.818
#1 and #2	86.7% (13/15)	80% (12/15)	0.855

*#1, Breast oncologist #1; #2, Breast oncologist #2*.

**Figure 4:**
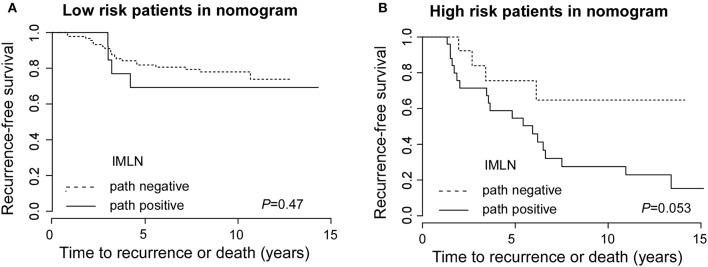
Recurrence-free survival for low risk patients with true negative and false negative groups and for high risk patients with true positive and false positive IMLN groups. Recurrence-free survival for low risk patients **(A)** with true negative and false negative groups and for high risk patients **(B)** with true positive and false positive IMLN groups.

Moreover, to evaluate the clinical impact of the FN and TN in an alternative way, we performed survival analyses comparing recurrence-free survival (RFS) based on nomogram and pathology results (Figures 4A,B). The RFS between FN-LR-nomogram and TN-LR-nomogram patients was equivalent (*P* = 0.47 or *P* = 0.053). We performed the cox regression analysis and found that only adjuvant radiotherapy can affect the patients' survival (Supplementary Table 2), however, in low-risk or high-risk patients (stratified by nomogram model), adjuvant radiotherapy cannot affect patients' prognosis (Supplementary Table 3). For low risk patients in nomogram, the similar survival status may be attributed to the similar treatment decision actually making in this two different subgroups, though the pathologically IMLN status is not the same. For high risk patients in nomogram, one possible reason for this result is that FN patients usually have adverse risk factors which can reduce the effectiveness of adjuvant treatment. Then, in this study, false classification by nomogram does not significantly affect treatment strategy and patient prognosis.

## Discussion

Though there are new data to support IMLN treatment, it is recognized that the optimal subgroups of patients with BC have not yet been clearly agreed on by experts (1, 7, 8). Hence, we must carefully use several risk factors to decide whether IMLN treatment is warranted, including patient age, individual anatomy, tumor location, number, and volume of axillary lymph nodes metastases, histopathological features, cardiopulmonary complications, and perhaps life expectancy (1, 9–12). However, oncologists cannot identify patients who may benefit from IMLN radiotherapy or operation in the era of precision medicine. Therefore, accurate prediction assessment for IMLN status is essential for the selection of appropriate therapy (13–16). The main purpose of our analysis was to evaluate the value of clinical markers and various widely available biomarkers for predicting the IMLN status. In this analysis of retrospectively collected single center data, we identified IMLN metastasis-related risk factors, such as tumor location, LVI and axillary lymph node status, and then constructed a novel nomogram model based on the currently available predictor variables. We constructed the clinical-related factors-based nomogram model because the risk factor data are available for patients with BC, and this may make the model able to be widely applied as an interactive risk prediction tool.

Some studies reported age under 35 years as a risk factor for IMLN involvement (3). Many papers have also showed that positivity of axillary lymph nodes is the strongest predictive factor for IMLN involvement (2, 8, 17). Tumors with a medial location and larger size are associated with a higher rate of IMLN metastasis as well (18, 19). However, compared with previous investigations, age and tumor size were not predictive for IMLN metastasis in our present analysis. Previous studies have demonstrated that PET/CT and MRI are superior to conventional diagnostic techniques for detection of IMLN metastases (20–22). Contrary to others' findings, we conducted a more comprehensive research on the clinical-related factors and found PET/CT or MRI is not an independent predictive variable for IMLN metastasis.

As is known to all, current controversies about the selection of patients for regional nodal treatment remain, but there is general consensus and acceptance of the fact that the risks and benefits of regional nodal irradiation should be discussed and considered in appropriately selected patients (23–25). Radiotherapy to the IMLN is among the most controversial and polarizing issues in radiation oncology, owing to conflicting data on potential outcome benefits and cardiopulmonary toxic effects. Previous studies have also found an increase in cardiac mortality for patients treated with radiation therapy for left-sided breast cancers compared with right-sided breast cancers (26). To overcome the disadvantage of radiotherapy, our colleagues designed a novel technique to perform IMLNs operation by video-assisted thoracoscopic surgery (VATS) (5). They have demonstrated that VATS IMLNs operation is a minimally invasive surgical procedure and may provide more accurate staging for breast cancer patients. Moreover, the operation procedure is well-tolerated and the length of stay or morbidity is not increased. We then conducted IMLN operation by VATS method and evaluate the role of clinical factors on IMLN metastasis prediction in BC patients.

Currently, anatomic staging is steadily decreasing in importance for BC patients' adjuvant treatment decision making, our results of the blinded reviewers' treatment recommendations further support the concept (27). Some of the inconsistency in adjuvant treatment recommendations between two reviewers may be due to the guideline debate about chemotherapy and radiotherapy. Oncology adjuvant treatment decision making may be influenced by past, present and future as well. As our expected, there was no significant difference in RFS for patients with a FN nomogram compared to a TN nomogram, suggesting that if nomogram misses IMLN disease, it is likely to be insignificance in clinical practice. However, the FN rate of nomogram for the detection of IMLN disease was 13.9%, which is higher than the FN rate of sentinel lymph node biopsy (<10%).

There are also some limitations to our current study. Firstly, it was a retrospective study with small sample sizes. Secondly, the enrolled patients with BC didn't match with their molecular subtype. Thirdly, the study was conducted on a population of more than 50% pT2 patients and about 10% pT3, though these patients with pT3 were probably candidated to radiation therapy of the chest wall, regardless of IMLNs status. Fourthly, there wasn't independent external validation cohorts from other hospitals in our current study. Therefore, prospective, large-scale and multicenter clinical trials should be carried out in future.

## Conclusion

To sum up, an objective and accurate prediction nomogram model for IMLN metastases was drawn up and validated in patients with BC. The new established nomogram model, as a robust tool in predicting IMLN involvement, has proved easy to use and sufficiently accurate to predict the IMLN metastasis risk in women with BC. Furthermore, it was able to select patients at high risk of IMLN metastasis to plan appropriate treatment strategies, while omission of IMLN surgery or radiotherapy for low risk patients.

## Data Availability Statement

The datasets used and analyzed during the current study are available from the corresponding author on reasonable request.

## Ethics Statement

This study was approved by the Ethics Committees of Sun Yat-sen University Cancer Center, and conducted in accordance with the Helsinki Declaration. Informed consent was obtained from all patients included in the study.

## Author Contributions

XinX, ZX, and XiaX conceived the experiments. XinX, HT, and XH conducted the experiments. XL and ZX analyzed and interpreted the data. XinX and FY wrote the manuscript. XinX and ZX prepared the figures. All authors read and approved the final manuscript.

### Conflict of Interest

The authors declare that the research was conducted in the absence of any commercial or financial relationships that could be construed as a potential conflict of interest.
