# Supplementary material for: Nomogram to Predict Internal Mammary Lymph Nodes Metastasis in Patients With Breast Cancer
Source: Front Oncol. 2019 Nov 8;9:1193. doi: 10.3389/fonc.2019.01193 (PMC6857087; doi:10.3389/fonc.2019.01193)
Supplement: Supplementary file 1 [file Table_1.DOC]

**Supplementary Figure 1: Blinded review comparing adjuvant systemic therapy recommendations made by two medical oncologists versus actual systemic therapy given in patients with low risk by nomogram**

| **False Negative nomogram** | | |  | **Matched True Negative nomogram** | | |
| --- | --- | --- | --- | --- | --- | --- |
| Actual | Reviewer #1 | Reviewer #2 |  | Actual | Reviewer #1 | Reviewer #2 |
|  |  |  |  |  |  |  |
|  |  |  |  |  |  |  |
|  |  |  |  |  |  |  |
|  |  |  |  |  |  |  |
|  |  |  |  |  |  |  |
|  |  |  |  |  |  |  |
|  |  |  |  |  |  |  |
|  |  |  |  |  |  |  |
|  |  |  |  |  |  |  |
|  |  |  |  |  |  |  |
|  |  |  |  |  |  |  |
|  |  |  |  |  |  |  |
|  |  |  |  |  |  |  |
|  |  |  |  |  |  |  |
|  |  |  |  |  |  |  |

**Legend:**

Black: Chemotherapy, radiotherapy and endocrine therapy

Blue: Chemotherapy and endocrine therapy

Yellow: Endocrine therapy

Green: Chemotherapy, herceptin and radiotherapy therapy

Gray: Chemotherapy, herceptin, radiotherapy therapy and endocrine therapy

Purple: Chemotherapy, herceptin and endocrine therapy

Orange: Chemotherapy and radiotherapy therapy

Red: Chemotherapy and herceptin

**Supplementary Table 1: Demographic and clinicopathologic characteristics of blinded review patients in Group 1 (False Negative low risk nomogram) and Group 2 (True Negative low risk nomogram)**

| **Characteristics** | **Group 1**  **(n = 61)**  **n (%)** | **Group 2**  **(n= 61)**  **n (%)** | **p-value** |
| --- | --- | --- | --- |
| Age (years) |  |  | 0.066 |
| Median (range) | 53 (31-62) | 46.6 (25-68) |  |
| Tumor size |  |  | 0.180 |
| T1 | 6 (40) | 2 (13.3) |  |
| T2 | 9 (60) | 12 (80) |  |
| T3 | 0 (0) | 1 (6.7) |  |
| Tumor location |  |  | 0.339 |
| UIQ  LIQ  Central  UOQ  LOQ | 9 (60) | 6 (40) |  |
| LIQ  LIQ  Central  UOQ  LOQ | 2 (13.3) | 1 (6.7) |  |
| Central  LIQ  Central  UOQ  LOQ | 0 (0) | 1 (6.7) |  |
| UOQ  LIQ  Central  UOQ  LOQ | 33 (20) | 7 (46.7) |  |
| LOQ  LIQ  Central  UOQ  LOQ | 1 (6.7) | 0 (0) |  |
| ER status |  |  | 0.361 |
| Positive | 11 (73.3) | 13 (86.7) |  |
| Negative | 4 (26.7) | 2 (13.3) |  |
| Unknown | 0 (0) | 0 (0) |  |
| PR status |  |  | 1.0 |
| Positive | 10 (66.7) | 10 (66.7) |  |
| Negative | 5 (33.3) | 5 (33.3) |  |
| Unknown | 0 (0) | 0 (0) |  |
| Her2 status |  |  | 0.183 |
| Positive | 4 (26.7) | 2 (13.3) |  |
| Negative | 9 (60) | 13 (86.7) |  |
| Unknown | 2 (13.3) | 0 (0) |  |
| LVI |  |  | 1.0 |
| Yes | 0 (0) | 0 (0) |  |
| No | 15 (100) | 15 (100) |  |
| pN stage |  |  | 0.673 |
| N0 | 6 (40) | 4 (26.7) |  |
| N1 | 8 (53.3) | 9 (60) |  |
| N2 | 1 (6.7) | 2 (13.3) |  |
| N3 | 0 (0) | 0 (0) |  |
| NAC received |  |  | 0.759 |
| Yes | 1 (6.7) | 1 (6.7) |  |
| No | 14 (93.3) | 14 (93.3) |  |
| Imaging-reported IMLN status |  |  | 0.283 |
| Positive | 3 (20) | 1 (6.7) |  |
| Negative | 12 (80) | 14 (93.3) |  |

ER, estrogen receptor; PR, progesterone receptor; Her2, human epidermal growth factor receptor-2; UIQ: upper inner quadrant; LIQ: lower inner quadrant; UOQ: upper outer quadrant; LOQ: lower outer quadrant; LVI: lymphvascular invasion; LVI: lymph vascular invasion; NAC: Neoadjuvant chemotherapy.

**sTable 2. Cox regression analysis for pIMLN status and treatment-related factors associated with recurrence-free survival (RFS)**

| **Characteristics** | **Univariate cox regression analysis** | |
| --- | --- | --- |
|  | HR(95%CI) | *P* value |
| pIMLN status |  | 0.007 |
| Negative | 1 |  |
| Positive | 2.445(1.280-4.671) |  |
| Adjuvant chemotherapy |  | 0.969 |
| No | 1 |  |
| Yes | 1.041(0.142-7.624) |  |
| Adjuvant radiotherapy |  | 0.008 |
| No | 1 |  |
| Yes | 3.039(1.334-6.922) |  |
| Adjuvant hormone therapy |  | 0.754 |
| No | 1 |  |
| Yes | 1.124(0.541-2.334) |  |

**sTable 3. Cox regression analysis for Adjuvant radiotherapy associated with associated with recurrence-free survival (RFS) in low- vs high-risk group patients**

| **Characteristics** | **Low-risk group patients** | | **High-risk group patients** | |
| --- | --- | --- | --- | --- |
|  | HR(95%CI) | *P* value | HR(95%CI) | *P* value |
| Adjuvant chemotherapy |  | 0.898 |  | 0.745 |
| No | 1 |  | 1 |  |
| Yes | 1.161(0.119-11.325) |  | 1.007(0.175-12.156) |  |
| Adjuvant radiotherapy |  | 0.171 |  | 0.919 |
| No | 1 |  | 1 |  |
| Yes | 2.121(0.723-6.222) |  | 1.125(0.116-10.911) |  |
| Adjuvant hormone therapy |  | 0.553 |  | 0.922 |
| No | 1 |  | 1 |  |
| Yes | 1.470(0.411-5.251) |  | 0.953(0.363-2.502) |  |
| pIMLN status |  | 0.812 |  | 0.666 |
| Negative | 1 |  | 1 |  |
| Positive | 1.176(0.310-4.460) |  | 1.331(0.364-4.872) |  |
